# Mild Heat Stimulating and Microenvironment Reprogramming Hydrogel for Accelerating Diabetic Wound Healing

**DOI:** 10.3390/gels12060542

**Published:** 2026-06-17

**Authors:** Xueting Xiao, Yannan Liu, Dan Li, Lebin Wang, Zirui Hu, Xinliang Xing, Yali Ding, Xurun Wang, Ruifan Zhang, Jing Yang, Xiaoxuan Ma

**Affiliations:** 1 Engineering Research Center of Western Resource Innovation Medicine Green Manufacturing, Ministry of Education, School of Chemical Engineering, Northwest University, Xi’an 710127, China; 2 Shaanxi Key Laboratory of Biomaterials and Synthetic Biology, Shaanxi R&D Center of Biomaterials and Fermentation Engineering, School of Chemical Engineering, Northwest University, Xi’an 710127, China

**Keywords:** diabetic wound healing, microenvironment reprogramming, mild photothermal stimulation, natural bioactive compounds, green synthesis

## Abstract

Diabetic wounds are characterized by persistent hyperglycemia, excessive ROS accumulation, sustained inflammation, and impaired angiogenesis, yet current treatments remain suboptimal. To address these challenges, we developed a mild heat stimulating and microenvironment reprogramming hydrogel (termed C-4-N) via a green synthetic strategy. L-Arginine (L-Arg) triggered the spontaneous self-polymerization of protocatechuic aldehyde (PA) into poly (protocatechuic aldehyde) (PPA) nanoparticles, onto which ginsenoside Compound K (CK) was subsequently loaded, yielding CK/L-Arg/PPA nanoparticles. These nanoparticles were then uniformly embedded into a dynamic disulfide network composed of α-lipoic acid (LA)-modified chitosan (CS-LA) and 4-arm-PEG-SH under UV irradiation without toxic photo-initiators, forming the C-4-N hydrogel. The C-4-N hydrogel reprogrammed the diabetic wound microenvironment through three synergistic mechanisms, lowering blood glucose and scavenging ROS via the coordinated actions of LA, CK and PPA, promoting M1-to-M2 macrophage polarization via downregulation of pro-inflammatory cytokines (TNF-α, IL-6) and upregulation of anti-inflammatory cytokines (IL-10, TGF-β1), further amplified by mild photothermal stimulation of 40–43 °C. In a diabetic rat model, the C-4-N hydrogel achieved a near-complete wound closure rate of 99.49 ± 0.10% on day 13 upon mild photothermal stimulation, accompanied by enhanced re-epithelialization, organized collagen deposition, vascular maturation, and systemic glucose regulation. In summary, this green synthesized, mild heat-stimulating hydrogel establishes a synergistic microenvironment reprogramming paradigm for chronic diabetic wound managements.

## 1. Introduction

Diabetes affects approximately 540 million people worldwide, and nearly 20% of diabetic patients develop chronic non-healing wounds [1], such as diabetic foot ulcers (DFUs), which often lead to lower limb amputation and increased mortality, imposing substantial socioeconomic burdens [2]. The pathophysiology of diabetic wounds is multifactorial, involving persistent hyperglycemia, excessive reactive oxygen species (ROS) production, prolonged inflammation, impaired angiogenesis, and peripheral neuropathy [3]. Despite advances in wound care management, including debridement, infection control, negative pressure, and growth factor therapy, current clinical treatments remain suboptimal due to unidimensional function, limited efficacy, high recurrence rates, and significant healthcare costs [4]. Several hydrogel systems loaded with cytokines, extracellular matrix components, or extracellular vesicles have been approved for promoting the healing of diabetic wounds [5,6]. However, these methods are costly, focus solely on monotherapy, and have limited efficacy. Therefore, the development of a comprehensive therapeutic approach that simultaneously alleviates oxidative stress, modulates chronic inflammation and promotes angiogenesis is crucial for the treatment of diabetic wounds [7].

Accumulating evidence indicates that the intractability of diabetic wounds primarily stems from a disrupted endogenous microenvironment driven by persistent hyperglycemia [8]. Chronically elevated blood glucose levels promote ROS overproduction through glucose auto-oxidation, protein glycation, and activation of the polyol pathway [9,10]. The resultant oxidative stress not only directly damages cellular components, such as lipids, proteins, and DNA [11], but also sustains pro-inflammatory signaling cascades [12], thereby delaying the transition from the inflammatory phase to the proliferative phase and perpetuating a chronic, non-healing state [13]. Macrophages, as central regulators of tissue repair, exhibit remarkable phenotypic plasticity. However, under diabetic conditions, the prolonged hyperglycemic and oxidative microenvironment disrupts the delicate balance between pro-inflammatory M1 and pro-healing M2 macrophages, leading to sustained M1-dominant polarization [14]. This imbalance perpetuates chronic inflammation, impairs apoptotic cell clearance, and compromises angiogenesis, all of which collectively hinder diabetic wound healing [15]. Consequently, therapeutic strategies that simultaneously lower blood glucose, scavenge excessive ROS, promote M1-to-M2 macrophage polarization, and enhance angiogenesis represent a promising paradigm for reprogramming the diabetic wound microenvironment and accelerating the healing process.

Given the multifaceted pathophysiology of diabetic wounds, a combination of natural bioactive compounds with synergistic mechanisms offers a promising strategy. To this end, four complementary agents, α-lipoic acid (LA), ginsenoside Compound K (CK), L-Arginine (L-Arg), and protocatechuic aldehyde (PA), collectively achieve glucose regulation, oxidative stress relief, immune reprogramming, and vascular regeneration. The therapeutic strategy is built on three synergistic pillars. First, to correct hyperglycemia and alleviate oxidative stress, LA enhances insulin sensitivity via the PI3K/Akt/GLUT4 axis [16], while CK stimulates insulin secretion and protects pancreatic β-cells [17]. Concurrently, LA directly scavenges ROS through its thiol-disulfide cycle [18], and the catechol-rich PA/PPA provides additional antioxidant capacity via radical scavenging [19], metal chelation, and Nrf2 pathway activation [20]. Second, to reprogram immune microenvironment and resolve inflammation, both LA and CK converge to promote M1-to-M2 macrophage polarization by suppressing NF-κB-driven pro-inflammatory cytokines [21,22] (TNF-α, IL-6) and upregulating anti-inflammatory mediators (IL-10, TGF-β1). Meanwhile, L-Arg exerts direct immunomodulatory effects by upregulating PRDX1 expression to maintain mitochondrial homeostasis, and regulating the iNOS/Arg-1 metabolic balance, thereby further promoting M2 polarization [23]. Third, to promote angiogenesis and tissue repair, LA not only generates pro-angiogenic H_2_S upon disulfide cleavage, but also enhances eNOS activity [24]. L-Arg serves as the substrate for eNOS to produce NO [25], and the LA-derived H_2_S further amplifies this pathway by activating PI3K/Akt/eNOS signaling [26]. The resulting H_2_S-NO crosstalk not only drives robust angiogenesis but also reinforces M2 polarization [27,28], creating a pro-regenerative microenvironment. Thus, this rationally designed combination of natural compounds acts in concert to simultaneously lower blood glucose, scavenge ROS, reprogram macrophage polarization and promote angiogenesis.

In parallel, mild heat stimulation has been established as an effective physical therapy for wound healing. Recent studies have confirmed that mild heat treatment (40 and 41 °C) promotes the conversion of M1 macrophages to M2 macrophages and enhances angiogenesis [29]. Preclinical studies have demonstrated that local hyperthermia of 40–44 °C promotes endothelial cell tube formation and enhances neovascularization in ischemic tissues [30]. Notably, PPA, derived from the self-polymerization of PA, exhibits efficient photothermal conversion under NIR irradiation [31], and the mild heat it generates could be tuned to remain within the 40–44 °C range, thereby enhancing angiogenesis [32] and tissue repair without causing thermal damage to surrounding healthy tissues [33]. Furthermore, in line with our green chemistry approach, L-Arg not only serves as a substrate for pro-angiogenic NO production but also creates a mild alkaline environment that spontaneously triggers the self-polymerization of PA into PPA [34]. Thus, the integration of L-Arg and PPA provides a green, self-contained platform that simultaneously scavenges ROS, generates a pro-angiogenic NO environment and enables controlled mild photothermal stimulation to accelerate diabetic wound healing [35].

**Scheme 1:**
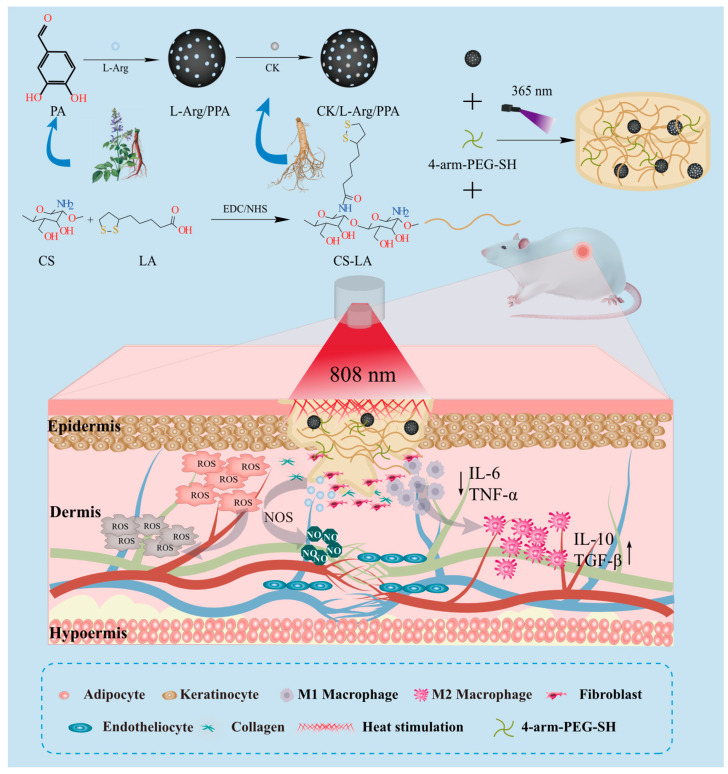
Schematic illustration of the green-synthesized, mild heat-stimulating C-4-N hydrogel for diabetic wound healing.

Based on the above considerations, we developed a mild heat-stimulating and microenvironment-reprogramming hydrogel (termed C-4-N) via a green synthetic strategy for accelerating diabetic wound healing (Scheme 1). The hydrogel integrated CK/L-Arg/PPA nanoparticles into a dynamic disulfide network of CS-LA and 4-arm-PEG-SH under UV irradiation, this hydrogel integrates CK/L-Arg/PPA nanoparticles into a dynamic disulfide network composed of CS-LA and tetra-branched PEG-SH, without the need for toxic initiators. In contrast, photo crosslinked hydrogels reported in the literature typically require the addition of photo initiators such as I2959 or LAP, which pose a potential risk of cellular damage [36], which constitutes a key advantage of the photo crosslinked hydrogel in this study over other hydrogels. The self-polymerization of PA is spontaneously triggered by the basic conditions induced by L-Arg. By replacing the traditional harsh process with milder conditions, we have improved the controllability of the polymerization reaction. Unlike previously reported multifunctional hydrogels for diabetic wounds (which primarily incorporate modules such as antioxidant [37], anti-inflammatory, or angiogenic properties), the C-4-N hydrogel can remodel the microenvironment of diabetic wounds through three synergistic mechanisms: lowering blood glucose levels, scavenging reactive oxygen species (ROS), promoting the polarization of macrophages from the M1 to the M2 phenotype, and enhancing angiogenesis. All of these effects are achieved through the synergistic interaction of natural bioactive compounds and mild photothermal stimulation, rather than through the simple combination of bioactive components. Collectively, this green-synthesized, mild heat stimulating hydrogel establishes a synergistic microenvironment reprogramming therapeutic paradigm, offering a promising and translatable strategy for chronic diabetic wound management.

## 2. Results and Discussion

### 2.1. Synthesis and Characterization of the Composite Hydrogel

**Figure 1:**
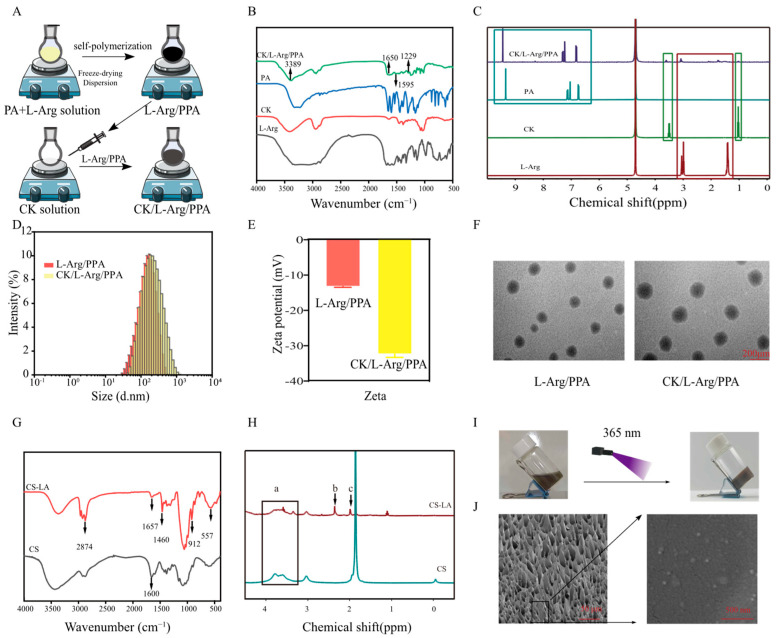
Synthesis of C-4-N composite hydrogels. (**A**) Synthesis procedure of the CK/L-Arg/PPA nanoparticles. FT-IR spectra (**B**), ^1^H NMR spectra (**C**), Particle size (**D**), Zeta potential (**E**), and TEM image (**F**) of the CK/L-Arg/PPA nanoparticles. FT-IR spectra (**G**) and ^1^H NMR (**H**) spectra of CS-LA. (**I**) Photograph of the C-4-N hydrogel formation under UV of 365 nm. (**J**) SEM of the C-4-N composite hydrogel.

CK/L-Arg/PPA nanoparticles synthesis procedure was shown in Figure 1A. PA was spontaneously and oxidatively self-polymerized into PPA in the L-Arg induced basic condition. Then, CK was loaded into L-Arg/PPA via hydrophobic interactions and hydrogen bonding, forming the CK/L-Arg/PPA nanoparticles.

The chemical structures of the synthesized CK/L-Arg/PPA nanoparticles were characterized by Fourier Transform infrared (FT-IR) spectroscopy (Figure 1B). Compared with free PA, the CK/L-Arg/PPA spectrum showed a characteristic C=O stretching vibration peak at 1650 cm^−1^, confirming the presence of carbonyl/aldehyde groups. A new absorption peak emerged at 1595 cm^−1^, corresponding to the -N=C- stretching vibration. This indicates the successful formation of a Schiff base linkage between the amino groups of L-Arg and the aldehyde group(s) of PPA. Additionally, a new peak at 1229 cm^−1^ was assigned to the characteristic -C-N- stretching vibration arising from the phenol-amine interaction between the phenolic hydroxyl groups of PPA and the -NH_2_ groups of L-Arg [34,38]. Notably, compared with other groups, the -OH stretching peak of CK/L-Arg/PPA shifted to a higher wavenumber of 3389 cm^−1^, suggesting the formation of hydrogen bonds, and indicating the successful loading of CK. ^1^H Nuclear magnetic resonance (^1^H NMR) further confirmed the preparation of the target CK/L-Arg/PPA nanoparticles, as evidenced by the retention of characteristic resonance signals from each component (Figure 1C). Specifically, the signal at 9.45 ppm was attributed to the -CHO of PPA, while the multiple peaks at 7.31, 7.23, and 6.83 ppm corresponded to the aromatic protons of PPA. Signals at 3.60 ppm and 1.02 ppm were assigned to the -OH and -CH_3_ protons of CK, respectively. Resonance peaks at 3.08 ppm and 1.75 ppm corresponded to the -CH- and -CH_2_- protons of L-Arg, respectively. The coexistence of these characteristic nuclear magnetic resonance signals confirmed, at the molecular level, that the CK/L-Arg/PPA nanoparticle system contains all three active components of CK, L-Arg, and PA. In addition, high-performance liquid chromatography was used in this study to determine the drug loading of CK, which was found to be 32.27 ± 0.10%, with an encapsulation efficiency of 47.67 ± 0.22%. Taken together, these data confirm the successful preparation of the target CK/L-Arg/PPA nanoparticles.

Dynamic light scattering (DLS) analysis revealed that the L-Arg/PPA nanoparticles exhibited a mean hydrodynamic diameters of176.62 ± 7.03 nm, while the CK/L-Arg/PPA nanoparticles showed a slightly larger size of 200.64 ± 12.86 nm (Figure 1D). The corresponding zeta potentials were −13.36 ± 0.20 mV and −32.5 ± 0.77 mV, respectively (Figure 1E). The relatively high absolute zeta potential of the CK/L-Arg/PPA nanoparticles suggests that they can resist aggregation through surface electrostatic repulsion, indicating good dispersion stability. Transmission electron microscopy (TEM) observations further verified that both formulations exhibited well-defined, monodisperse spherical morphologies (Figure 1F), consistent with the DLS results. To further investigate the stability of the nanoparticles under physiological conditions in this study, the nanoparticles were dispersed in PBS at pH 7.4, and their particle size and zeta potential were measured at 0, 24, and 48 h. As shown in Table S2, the particle size increased gradually and slightly with prolonged standing time, and the nanoparticle structure did not collapse within 48 h. Overall, the nanoparticles exhibited excellent stability within 24 h. Although stability declined somewhat by 48 h, no significant flocculation or sedimentation occurred.

The successful modification of CS with LA (CS-LA) was characterized using FT-IR and ^1^H NMR. As shown in Figure 1G, the characteristic amino absorption peak of CS at 1600 cm^−1^ disappeared after conjugation. Concurrently, CS-LA exhibited a distinct amide C=O stretching peak at approximately 1657 cm^−1^ and an aliphatic C-H stretching peak at 2874 cm^−1^, indicating successfully LA conjugation [39]. The peak at 1460 cm^−1^ was assigned to the bending vibration of -CH_2_, while the peak at 912 cm^−1^ corresponded to the stretching vibration of C-S bond within the ring structure. Additionally, the absorption peak at 557 cm^−1^ was attributed to the disulfide bond [40], further supporting the occurrence of the amidation reaction between the amino groups in CS and the carboxyl groups in LA. The ^1^H NMR spectrum provided additional confirmation (Figure 1H). Compared with parent CS, the CS-LA spectrum exhibited new proton signals in the range of 3.23 to 3.8 ppm (peak a), as well as two new peaks at 2.19 ppm (peak b) and 1.83 ppm (peak c), identifying as protons within the -CH_2_ group in LA. These ^1^H NMR data confirmed the successful preparation of CS-LA conjugate. Moreover, elemental analysis further revealed a grafting rate of 43.61% (Table S1). The C-4-N hydrogel was rapidly formed after mixing CS-LA, 4-arm-PEG-SH, and CK/L-Arg/PPA nanoparticles under UV irradiation (Figure 1I).

Scanning electron microscopy (SEM) revealed that the freeze-dried hydrogel exhibited a exhibited a three-dimensional porous network structure with uniform pore size. At higher magnification, CK/L-Arg/PPA nanoparticles were observed to be evenly distributed across the pore walls (Figure 1J). Collectively, these results confirm the successful fabrication of the C-4-N hydrogel. Its physicochemical and biological properties will be systematically characterized in the following sections.

### 2.2. Physicochemical Properties

**Figure 2:**
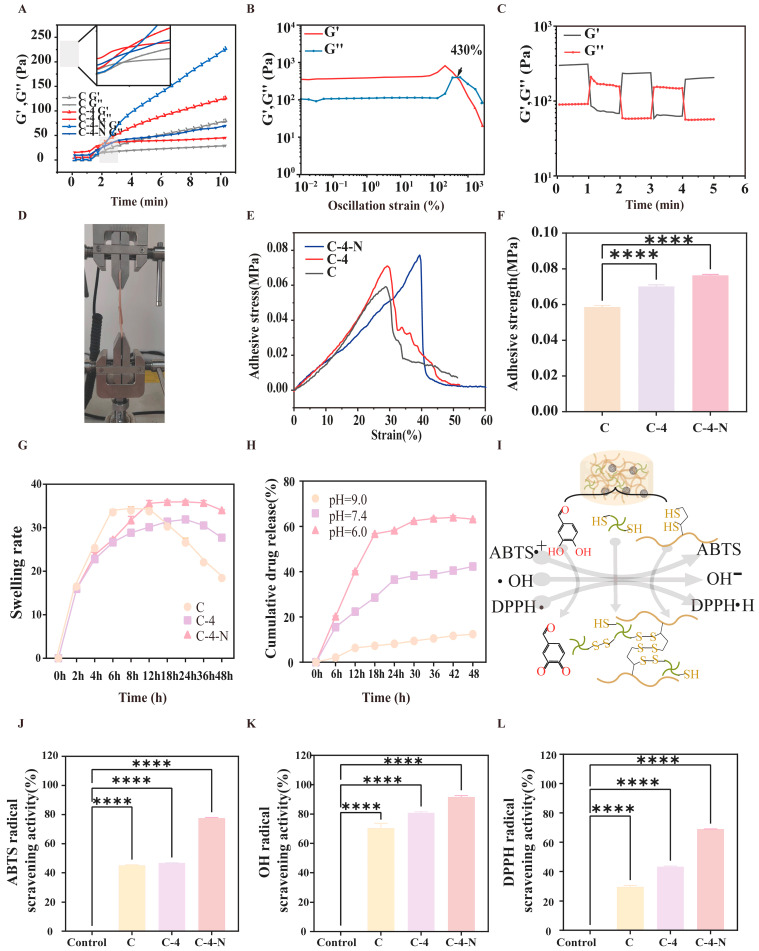
Characterizations of the composite hydrogels. Rheological behavior of the hydrogel: time-sweep (**A**), amplitude-sweep (**B**), and step-strain (**C**) tests. (**D**) Schematic illustration of the lap-shear adhesion strength test. Representative lap-shear stress–strain curves (**E**) and corresponding adhesive strength (**F**). (**G**) The swelling behavior of the composite hydrogels. (**H**) Cumulative drug release of C-4-N hydrogel at different pH. (**I**) Schematical illustration of ROS scavenging mechanisms. ROS scavenging efficiency of the composite hydrogels evaluated by ABTS (**J**), ·OH (**K**) and DPPH (**L**) radical assays. Sample size *n* = 3. **** *p* < 0.0001.

The rheological properties and self-healing behavior of the C-4-N hydrogel were systematically investigated. Upon UV irradiation, the disulfide bonds within the CS-LA underwent cleavage and subsequent random reformation, leading to the formation of a hydrogel network. As shown in Figure 2A, gelation occurred when the storage modulus (G′) exceeded the loss modulus (G″). The incorporation of 4-arm-PEG-SH provided additional free thiol groups for gelation, thereby enhancing the hydrogel strength. While further incorporation of CK/L-Arg/PPA nanoparticles rendered the precursor solution darker, reducing UV light transmittance and consequently slightly prolonging the gelation time from 49.8 s to 60 s. The macroscopic self-healing behavior of the hydrogel is shown in Figure S1. To facilitate visual tracking, one piece of the hydrogel was stained with rhodamine B appearing red, while the other piece remained transparent and colorless. When brought into contact at the freshly cut interface, the two pieces rapidly merged into a single, coherent hydrogel within seconds. The merged hydrogel could withstand manual manipulation and maintained its structural integrity upon stretching. Notably, mutual interpenetration and spreading of the two differently colored parts were clearly observed at the interface. Furthermore, small hydrogel fragments also reconnected upon contact. Collectively, these observations demonstrate the excellent macroscopic self-healing capability of the C-4-N hydrogel. To further investigate the mechanism of self-healing, strain amplitude sweep and cyclic strain amplitude sweep tests were performed. As shown in Figure 2B, the crossover points of G′ and G″ occurred at approximately 430% strain, indicating the collapse of the hydrogel network. Subsequently, the self-healing property of the C-4-N hydrogel was quantitatively evaluated via a cyclic strain amplitude sweep test, which alternated between low strain (1%) and high strain (1000%). As depicted in Figure 2C, the C-4-N hydrogel exhibited rapid recovery of G′ upon returning to low strain conditions, confirming its excellent self-healing capability. In addition, a gradual decrease in the recovered G′ and G″ values were observed over successive cycles. This phenomenon is likely attributable to incomplete reformation of the dynamic disulfide bond network following each high-strain disruption cycle, which is consistent with observations in other self-healing hydrogels based on dynamic disulfide bonds [41]. Collectively, the C-4-N hydrogel exhibits rapid UV-triggered gelation and excellent self-healing behavior.

The adhesive performance of the composite hydrogels was systematically and quantitatively investigated. As shown in Figure 2D, the shear adhesive force of the composite hydrogels was measured using lap shear strength experiments. The incorporation of 4-arm-PEG-SH increased the adhesive strengths from 58.63 ± 0.55 kPa for the C hydrogel to 70.13 ± 0.80 kPa for the C-4 hydrogel, while the maximum strain remained nearly unchanged at around 29% (Figure 2E,F). This enhancement is attributable to the increased cohesive force imparted by 4-arm-PEG-SH within the C-4 hydrogel network. Notably, the C-4-N hydrogel exhibited an even higher adhesive stress of 76.36 ± 0.40 kPa, with the maximum strain extended to more than 39% (Figure 2E,F). The superior adhesion stress of the C-4-N hydrogel is primarily attributed to the combined adhesive and cohesive forces contributed by the CK/L-Arg/PPA nanoparticles, which enhance energy dissipation and cohesive strength within the hydrogel network. Additionally, the organ adhesion performance of the C-4-N hydrogel was also evaluated using a vertical suspension method, and the results are presented in Figure S2. In this experiment, five representative types of fresh ex vivo tissues, namely heart, liver, spleen, lung, and kidney, were selected as model substrates to assess the versatility of the hydrogel’s adhesion. After applying the hydrogel onto the surface of each tissue, the adhesion stability was examined. The results demonstrated that the C-4-N hydrogel adhered firmly to all tested moist tissue surfaces without peeling off or detaching under its own weight. These findings indicate that the C-4-N hydrogel possesses strong and universal adhesion capability across a wide range of organ tissues.

Swelling behavior is a key parameter for hydrogels using as wound dressings, as it directly influences exudate management, oxygen permeability, and sustained drug release. The swelling behavior of the composite hydrogels was investigated by immersing in PBS solution (pH = 7.4). As shown in Figure 2G, the C hydrogel swelled rapidly, reaching a maximum swelling ratio of 33.54 ± 0.53 within 6 h. The C-4 hydrogel, due to the addition of 4-arm-PEG-SH, formed additional cross-linked with CS-LA, which significantly reduced the pore size of the hydrogel and increased the network density, resulting in a more compact structure that hindered water molecule penetration. Consequently, the C-4 hydrogel exhibited a lower swelling ratio, reaching a maximum of 31.91 ± 0.24 at 24 h. In contrast, the C-4-N hydrogel displayed the highest swelling rate, reaching 35.62 ± 0.51 at 12 h, with sustained swelling over 48 h. This enhanced swelling capacity is attributed to the hydrophilic nature of the CK/L-Arg/PPA nanoparticles, which facilitate water uptake. Therefore, the C-4-N hydrogel, with its balanced and sustained swelling behavior, represents a promising candidate for diabetic wound healing applications.

The in vitro drug release behavior of the C-4-N hydrogel was investigated under different pH conditions to simulate the dynamic wound microenvironment. As shown in Figure 2H, the composite hydrogel exhibited pronounced pH-sensitive drug release properties, which correlate well with the temporal progression of diabetic wound healing. Under acidic conditions (pH = 6.0), the drug release rate was the fastest, showing a rapidly increasing trend, with a cumulative release rate of 63.20 ± 0.05% at 48 h; under neutral conditions (pH = 7.4), the drug release rate was moderate, showing a sustained, slow upward trend, with a cumulative release rate of approximately 42.27 ± 0.25% at 48 h; whereas under alkaline conditions (pH = 9.0), drug release was significantly inhibited, with a cumulative release rate of only 12.38 ± 0.02% at 48 h, exhibiting distinct sustained-release characteristics. For diabetic wounds, prolonged inflammation often leads to lower pH at the wound [42]. This makes C-4-N hydrogel, which exhibit enhanced drug-release properties under low pH conditions, particularly advantageous for the treatment of diabetic wound healing. In this study, the rapid release of anti-inflammatory PA by the hydrogel under acidic conditions can remodel the immune microenvironment. The pH-dependent release behavior of PA observed in our system can be rationalized by the following considerations. On the one hand, in acidic solutions, the glycosidic bonds of chitosan are prone to cleavage, producing water-soluble low-molecular-weight CS-LA, which leads to rapid weight loss. In neutral solutions, the degradation of CS-LA primarily depends on the hydrolysis of the ester bonds in the polylactic acid side chains [43], and its degradation rate is relatively slow [44]; On the other hand, this is attributed to the instability of disulfide bonds: under acidic conditions, disulfide bonds readily break to form thiol groups, leading to hydrogel degradation [45]; under alkaline conditions, thiol groups are oxidized back into disulfide bonds, thereby slowing the hydrogel degradation rate and consequently reducing the nanoparticle release rate. Overall, the above experiments confirm that the C-4-N hydrogel exhibits pH-responsive CK/L-Arg/PPA nanoparticles release properties, giving it advantages in the treatment of diabetic wounds.

The rheological, self-healing, and adhesive properties of the C-4-N hydrogel collectively endow it with significant advantages as an advanced wound dressing. Its shear-thinning behavior allows for minimally invasive injection and conformal coverage of irregular wound beds. The dynamic disulfide bond network provides autonomous self-healing, ensuring long-term barrier function against bacterial invasion under mechanical stress. The catechol-rich CK/L-Arg/PPA nanoparticles endow the hydrogel with strong and universal tissue adhesion, enabling wet-tissue sealing to prevent fluid leakage. This synergistic combination provides a robust platform for sustained drug release and wound healing, significantly outperforming conventional passive wound dressings.

ROS scavenging assays were conducted to evaluate the antioxidant activities of composite hydrogels toward ABTS, OH, and DPPH radicals (Figure 2J–L). Across all three radicals, the scavenging efficiency consistently ranked as follows: C-4-N exhibited the highest activity, followed by C-4, while C showed the lowest. Specifically, the C-4-N hydrogel achieved scavenging rates of 77.57 ± 0.33% (ABTS), 91.60 ± 0.79% (·OH), and 68.97 ± 0.09% (DPPH), representing approximately 1.7 to 2.3-fold improvements over the C-4 hydrogel. These data collectively validate the potent radical scavenging capabilities of the hydrogels, with the C-4-N hydrogel exhibiting the highest antioxidant activity across all tested radicals. The remarkable antioxidant capacity of the C-4-N hydrogel can be attributed to the synergistic contributions of multiple components within the system. First, LA, one of the most effective naturally occurring antioxidants, scavenges various free radical species and interacts synergistically with other antioxidant substances. The free sulfhydryl groups (-SH) in LA serve as excellent redox-regulating molecules, which can rapidly form disulfide bonds by donating hydrogen atoms, thereby exerting potent antioxidant effects. Second, the polyphenolic structures present in the CK/L-Arg/PPA nanoparticles can directly react with ROS by donating hydrogen atoms or electrons, forming stable quinone structures and thereby terminating free radical chain reactions. Third, the guanidinium group in the L-Arg molecule is rich in lone-pair electrons, which can directly neutralize ROS, such as hydroxyl radicals (·OH) and superoxide anions (O_2_^−^), through electron transfer, effectively terminating free radical chain reactions and reducing oxidative damage. The proposed ROS scavenging mechanisms are schematically illustrated in Figure 2I. Collectively, these synergistic mechanisms endow the C-4-N hydrogel with excellent and broad-spectrum ROS scavenging activity, which is expected to alleviate oxidative stress, suppress excessive inflammation, thereby accelerating wound healing. The full wavelength ranges for each free radical scavenging assay are shown in Supplementary Figure S3.

### 2.3. Photothermal Performance and Near-Infrared Assisted Antibacterial Properties

**Figure 3:**
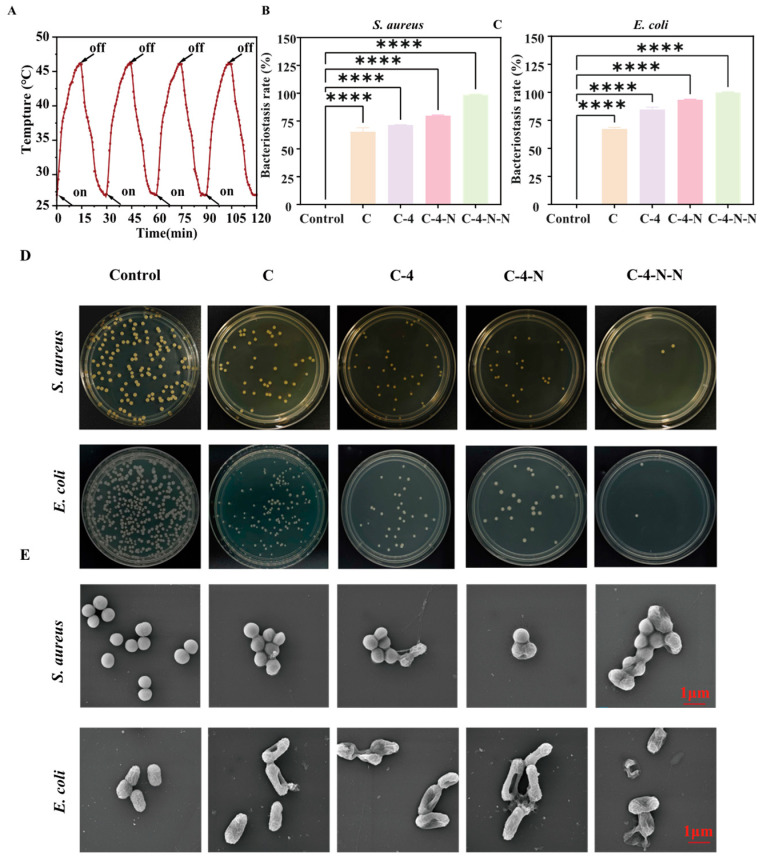
Photothermal and antibacterial properties of the composite hydrogels. (**A**) Photothermal heating curves of the C-4-N hydrogel. (**B**–**D**) Representative images and quantitative data in the antibacterial performance test against *S. aureus* and *E. coli*. (**E**) SEM images of the morphologies of *S. aureus* and *E. coli* after different treatments. Sample size *n* = 3. **** *p* < 0.0001.

To evaluate the photothermal effect of the composite hydrogels, it was irradiated with an 808 nm near-infrared (NIR) laser at a power density of 2.0 W cm^−2^ (Figure 3A). Upon irradiation, the hydrogel temperature gradually increased, reaching 46 °C after 15 min. To assess photothermal stability, the temperature changes were monitored over cycles consisting of 15 min of laser irradiation followed by 15 min of natural cooling. Notably, the composite hydrogel exhibited consistent and reproducible heating and cooling profiles throughout four consecutive on-off cycles, indicating excellent photothermal stability and reversibility. Importantly, the maximum temperature of 46 °C falls within the safe range for surrounding healthy tissues [46]. According to the investigation and the research, moderate local heat (45–50 °C) can effectively kill bacteria without causing damage to normal tissue cells [47]. Therefore, the photothermal effect of the C-4-N-N group, which is the C-4-N hydrogel under NIR irradiation, at 46 °C is expected to exert potent antibacterial activity while minimizing collateral damage to surrounding tissues, providing a strong rationale for the subsequent evaluation of its antibacterial performance.

The antibacterial performance of the composite hydrogels was determined through direct contact with bacterial suspensions followed by plate counting. As shown in Figure 3B–D, the antibacterial rates of the C hydrogel against *Escherichia coli* (*E. coli*) and *Staphylococcus aureus* (*S. aureus*) were 67.34 ± 1.26% and 65.37 ± 3.57%, respectively. This antibacterial effect is primarily attributed to the positively charged chitosan, which electrostatically interacts with negatively charged bacterial cell membranes, thereby disrupting membrane integrity, increasing permeability [48]. Additionally, the grafted LA exerted additional bactericidal effects through a dynamic disulfide and thiol equilibrium, inducing membrane depolarization, metabolic disruption, DNA damage, ultimately leading to bacterial death [49]. The additional thiol groups (-SH) introduced by 4-arm-PEG-SH in the C-4 hydrogel further enhanced the redox imbalance within bacterial cells, accelerating oxidative injury [50]. The C-4-N hydrogel demonstrated even stronger antibacterial activity, which is ascribed to the presence of PA. PA has been reported to inhibit ATP synthase and decrease intracellular adenosine triphosphate (ATP) levels in both Gram-positive and Gram-negative strains, thereby inhibiting bacterial growth and reproduction [51]. The strongest antibacterial capacity was exhibited by the C-4-N-N group. The localized photothermal effect caused irreversible, lethal damage to the bacteria, including membrane disruption, protein denaturation, and enzyme inactivation [52]. Together, these complementary mechanisms endow the C-4-N-N hydrogel with potent and broad-spectrum antibacterial activity.

To further visualize the antibacterial efficacy of the composite hydrogels, changes in bacterial morphology across different treatment groups were examined using SEM (Figure 3E). In the blank control group, both *S. aureus* and *E. coli* retained their intact native morphologies, displaying characteristic spherical and rod-shaped structures, respectively, with smooth and well-defined surfaces. For *S. aureus* treated with the C, C-4, and C-4-N hydrogels, some bacterial cells exhibited blurred edges, rough surfaces, and mild deformation, indicating initial membrane damage. In contrast, after treatment with the C-4-N-N hydrogel, *S. aureus* cells showed pronounced morphological alterations, with contours changing from spherical to shrunken, wrinkled, or collapsed states, indicative of severe membrane disruption and cell death. For *E. coli*, treatment with the C and C-4 hydrogels resulted in disrupted surface structures, with obvious shrinkage, indentations, and partial adhesion between adjacent cells. In the C-4-N group, *E. coli* cells exhibited leakage of intracellular contents, suggesting compromised membrane integrity. Strikingly, in the C-4-N-N group, the majority of *E. coli* cells underwent complete lysis, collapse, and structural disintegration, confirming the potent bactericidal effect of the combined chemical and photothermal treatment.

Collectively, these results demonstrate that the synergistic action of chemical components and NIR-assisted photothermal heating plays a crucial role in the antimicrobial process, effectively reducing the risk of wound infection and supporting subsequent tissue repair.

### 2.4. Biocompatibility, Cell Migration and Angiogenesis

**Figure 4:**
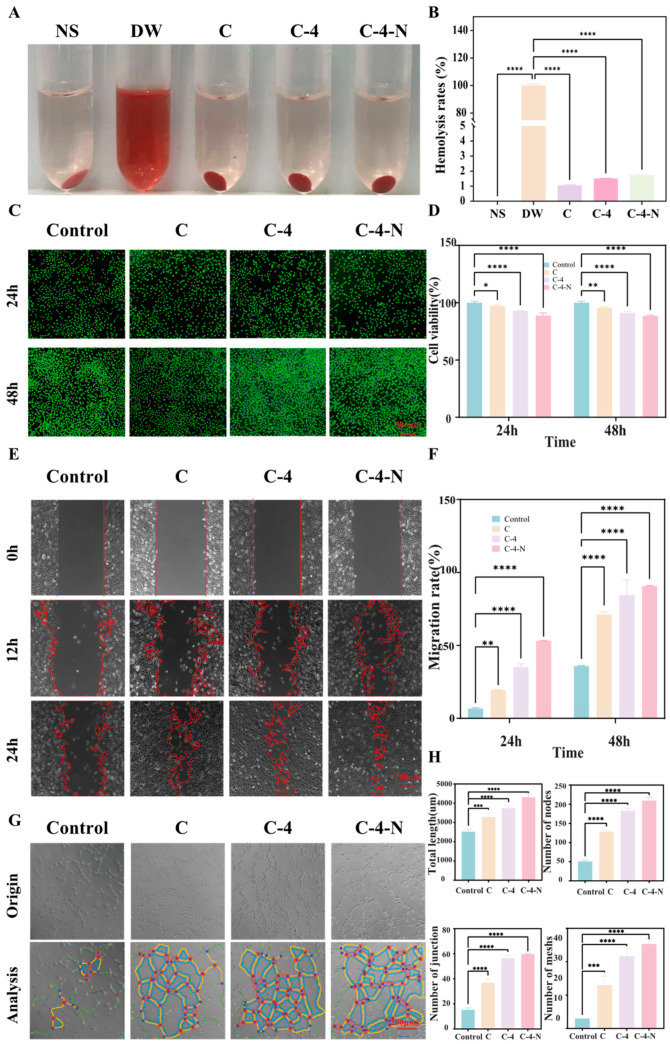
In vitro biocompatibility, cell migration, and angiogenic activity of the composite hydrogels. Hemolysis representative images (**A**) and quantitative rates (**B**). (**C**) Live/dead staining images and (**D**) cell viability of L929 cells after incubation for 24 and 48 h. (**E**) Representative images of cell migration at different time points (0, 12 and 24 h) and (**F**) the migration rate. (**G**) HUVECs tube formation images and (**H**) quantification analysis. Sample size *n* = 3. Red dots represent branch nodes (Junctions); yellow lines represent main vessel segments (Master Segments); green lines represent free branches (Branches); and blue closed areas represent the resulting mesh loops (Meshes). * *p* < 0.05, ** *p* < 0.01, *** *p* < 0.001, and **** *p* < 0.0001.

A fundamental requirement for hydrogels intended as clinical biomaterials is excellent biocompatibility. Hemolysis rate is one of the key criteria used to evaluate the blood compatibility of biomaterials. Based on hemolysis ratios, materials are classified into three categories: hemolytic (>5%), slightly hemolytic (2–5%), and nonhemolytic (<2%). As shown in Figure 4A,B, the hemolysis rates of the C, C-4, and C-4-N hydrogels were 1.06 ± 0.03%, 1.51 ± 0.01%, and 1.73 ± 0.34%, respectively, all below 5%. These results indicate that the hydrogels exhibit no significant hemolytic effects, possess good blood compatibility. As displayed in Figure 4D, no apparent cytotoxicity was observed in L929 cells after 24 h and 48 h of coincubation with hydrogel extracts. The cell viability in all hydrogel groups exceeded 85%, showing good biocompatibility of the composite hydrogels. Live/dead staining results further confirmed their favorable cytocompatibility, as evidenced by abundant live cells (green fluorescence) and rare dead cells (red fluorescence) across all groups (Figure 4C). Cell migration is an essential step in the wound healing process, as it facilitates the closure of the injured area. To evaluate the effect of the hydrogels on cell migration, a scratch wound healing assay was performed using human umbilical vein endothelial cells (HUVECs). As shown in Figure 4E,F, all hydrogel groups promoted cell migration compared with the control group. Quantitative data revealed that migration rates in experimental groups were significantly higher than in the control group. Notably, the C-4-N group exhibited the most pronounced migration-promoting effect, indicating its potential as a therapeutic dressing for diabetic wound healing. However, this study was conducted using standard culture medium; the limitations of this study should be taken into account in future research.

Endothelial tube formation is essential for angiogenesis in refractory wound repair, as newly formed blood vessels deliver oxygen and nutrients to regenerating tissues. Therefore, an in vitro tube formation assay was performed to evaluate the pro-angiogenic activity of the hydrogels. As shown in Figure 4G, all hydrogel-treated groups significantly promoted the formation of vascular branches and three-dimensional capillary-like networks, demonstrating potent angiogenic activity. Quantitative analysis (Figure 4H) revealed that the C-4-N group exhibited the strongest tube-forming capacity, featuring the highest number of nodes (209.66 ± 8.50) and junctions (59.50 ± 0.70) among all groups.

The enhanced cell migration and pro-angiogenic activity of the C-4-N hydrogel might be mechanistically attributed to the synergistic effects of hydrogen sulfide (H_2_S) signaling and redox microenvironment modulation. First, hydrogen sulfide (H_2_S), generated from the dynamic disulfide-thiol equilibrium of sulfur-containing components (including lipoic acid and 4-arm-PEG-SH), activates the PI3K/Akt and MAPK/ERK1/2 signaling pathways [53]. These pathways coordinate cytoskeletal reorganization, focal adhesion turnover, and endothelial cell proliferation [54], thereby driving cell migration and capillary-like tube formation. Second, the guanidinium-rich microenvironment provided by L-Arg, acts as an NO donor, continuously delivering pro-migratory and pro-angiogenic cues from the hydrogel matrix [55]. This sustained release stimulates both endothelial cell movement and new vessel formation, creating a self-amplifying regenerative loop that promotes wound healing. Third, the ROS-scavenging components of the hydrogel modulate mild oxidative stress, potentially preconditioning endothelial cells through activation of Nrf2, stress-responsive transcription factors [56]. This upregulates cytoprotective and angiogenic gene expression, enhancing migratory and tubulogenic capacity. Collectively, these synergistic mechanisms endow the C-4-N hydrogel with the ability to simultaneously promote cell migration and angiogenesis, making it highly effective for accelerating diabetic wound healing and tissue regeneration.

### 2.5. Antioxidant and Anti-Inflammatory Capacity

**Figure 5:**
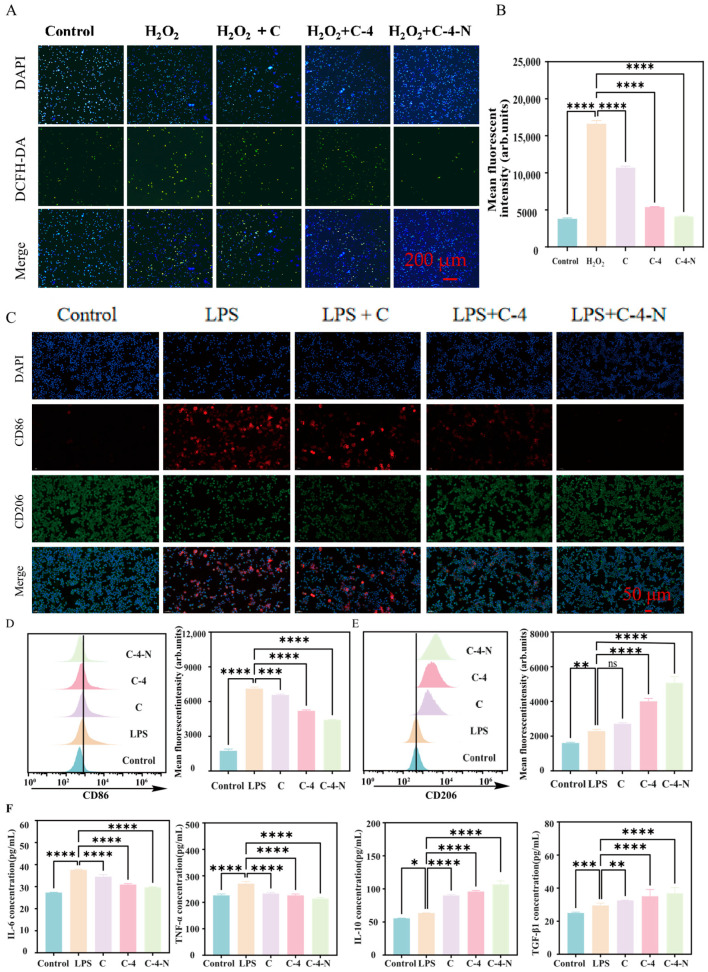
In vitro antioxidant and anti-inflammatory effects of the composite hydrogels. (**A**) Fluorescence images of ROS scavenging activity, and (**B**) quantitative fluorescence intensity. (**C**) Immunofluorescence staining of CD86 and CD206. Flow cytometry quantification of CD86-positive (**D**) and CD206-positive (**E**) populations. (**F**) ELISA quantification of pro-inflammatory (IL-6, TNF-α) and anti-inflammatory (IL-10, TGF-β1) cytokines. Sample size *n* = 3. * *p* < 0.05, ** *p* < 0.01, *** *p* < 0.001, and **** *p* < 0.0001.

Oxidative stress and inflammatory responses are key pathological factors that impede diabetic wound healing. Persistent hyperglycemia in diabetic patients leads to excessive production of reactive oxygen species (ROS), which not only directly damages cells but also triggers sustained inflammatory cascades, creating a detrimental cycle that disrupts normal wound healing. To verify the antioxidant capacity of the composite hydrogel, its intracellular ROS scavenging activity was investigated using the DCFH-DA probe. Upon cellular entry, the non-fluorescent DCFH-DA probe is deacetylated to DCFH by intracellular esterases and subsequently oxidized by ROS to highly fluorescent DCF, enabling both visual and quantitative assessment of intracellular ROS levels. As shown in Figure 5A, following stimulation with H_2_O_2_, the RAW264.7 cells exhibited strong green fluorescence, indicating elevated intracellular ROS levels and confirming the successful establishment of the oxidative stress cell model. In contrast, cells treated with the composite hydrogels showed markedly weaker fluorescence, demonstrating effective ROS scavenging. Notably, the C-4-N group exhibited the lowest fluorescence among all hydrogel-treated groups, suggesting that the incorporation of CK/L-Arg/PPA nanoparticles significantly enhances the antioxidant capacity. Furthermore, flow cytometry results quantitatively corroborated the fluorescence imaging observations (Figure 5B). The C-4-N group significantly reduced the fluorescence intensity, restoring it to a level comparable to that of the control group. These results collectively demonstrate that the C-4-N hydrogel possesses potent intracellular ROS scavenging activity and effectively protects cells from oxidative stress-induced damage.

Macrophages are key regulators of wound microenvironment, with M1 macrophages promoting inflammation and M2 macrophages mediating anti-inflammation and tissue repair. Thus, shifting the M1-to-M2 balance is a critical strategy for mitigating excessive inflammation in diabetic wounds. To evaluate the immunomodulatory capacity of the hydrogels, RAW 264.7 macrophages were first stimulated with LPS for 24 h to induce M1 polarization. As shown in Figure 5C, immunofluorescence staining revealed that LPS-treated cells exhibited strong red fluorescence (CD86, M1 macrophages marker) with minimal green fluorescence (CD206, M2 macrophages marker), confirming successful M1 polarization. In contrast, cells treated with the composite hydrogels showed markedly reduced red fluorescence and enhanced green fluorescence, indicating a shift from the M1 toward the M2 phenotype. Notably, the C-4-N group displayed the most pronounced effect. Flow cytometry analysis quantitatively confirmed these observations (Figure 5D,E). LPS stimulation dramatically increased the proportion of CD86 expression, whereas hydrogel treatment groups significantly reduced CD86 and increased CD206 expressions. The C-4-N group exhibited the highest M2/M1 ratio of 1.14 ± 0.06, indicating its potent capacity to reprogram macrophages from a pro-inflammatory to a pro-regenerative state. This quantitative evidence corroborates the immunofluorescence findings and underscores the synergistic contribution of the C-4-N hydrogel to immunomodulation. To further support the immunomodulatory effect, key pro-inflammatory and anti-inflammatory cytokines were also evaluated by ELISA (Figure 5F). Compared with the LPS group, hydrogel treatment decreased pro-inflammatory cytokines (IL-6, TNF-α) and increased anti-inflammatory cytokines (IL-10, TGF-β1). The C-4-N group consistently showed the most favorable cytokine profile. Collectively, these results demonstrate that the C-4-N hydrogel effectively promotes M1-to-M2 macrophage polarization, reduces pro-inflammatory cytokine secretion, and enhances anti-inflammatory cytokine production. This immunomodulatory capability shortens the inflammatory phase and accelerates diabetic wound healing.

### 2.6. Wound Healing

**Figure 6:**
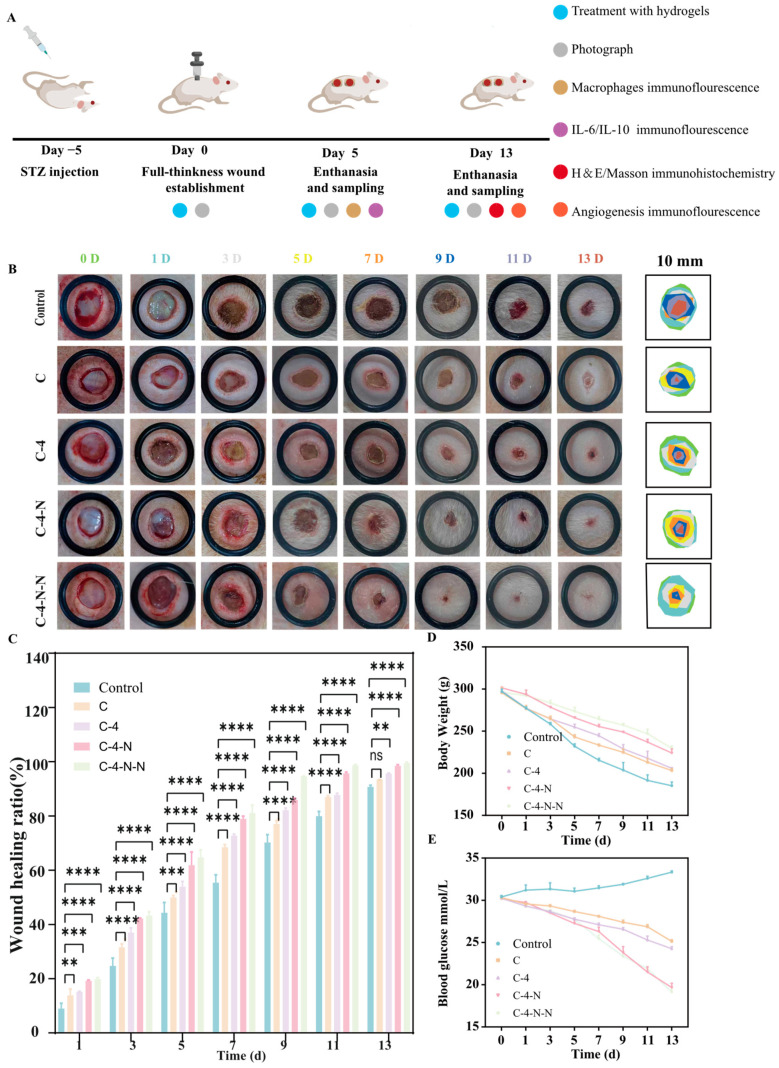
In vivo evaluation of the composite hydrogels in diabetic wound healing. (**A**) Experimental timeline of diabetic rat model establishment and treatment. (**B**) Representative wound images at different time points. (**C**) Wound healing rates in each group at different time points. (**D**) Body weight changes over time. (**E**) Blood glucose changes over time. Sample size *n* = 5. ns: not significant, ** *p* < 0.01, *** *p* < 0.001, and **** *p* < 0.0001.

A diabetes model was established in SD rats using streptozotocin (STZ). The progression of this study is detailed in Figure 6A. After maintaining blood glucose levels above 16.7 mmol/L for 5 days to confirm stable diabetes, full-thickness excisional wounds (diameter of 8 mm) were created on the dorsal skin of each rat and rats were randomly assigned to five groups, the untreated control, C hydrogel, C-4 hydrogel, C-4-N hydrogel, and C-4-N-N hydrogel groups (Each group consisted of ten SD rats. A total of 50 SD rats were used.). For the C-4-N-N group, the hydrogel was irradiated with an 808 NIR laser (1.8 W/cm^2^, 10 min) to reach a surface temperature of approximately 43 °C (Figure S4). This temperature range (39–43 °C) was selected based on previous reports that mild heat stimulation promotes cell migration, enhances angiogenesis, and accelerates wound closure without causing thermal damage to surrounding healthy tissues [57]. In addition, we conducted photothermal stability tests. As shown in Figure S4B, after each round of laser irradiation, the temperature rose steadily to the target photothermal range of approximately 43 °C; the peak temperatures from the three rounds showed high consistency, with no significant decline. Furthermore, considering that the dressing was replaced every other day in this study, we measured the photothermal temperatures at 0 and 1 days after dressing application. As shown in Figure S4C, the heating rates and peak temperatures at 0 and 1 days were highly consistent. This indicates that photothermal efficiency did not decline after 1 day, demonstrating that the hydrogel’s photothermal conversion performance remained stable. Wound healing was monitored on days 0, 1, 3, 5, 7, 9, 11, and 13 post-wounding to track the dynamic closure process across all experimental groups. As shown in Figure 6B,C, the C-4-N-N group consistently exhibited the most rapid wound closure throughout the observation period. By day 9, the C-4-N-N group had achieved a healing rate of 92.05%, whereas all other groups remained significantly below 90% at the same time point. Specifically, the healing rates of the control, C, C-4, and C-4-N groups on day 9 were 59.92%, 68.25%, 83.18%, and 84.57%, respectively. With continued treatment and extended observation, the C-4-N-N group ultimately achieved a near-complete healing rate of 99.49 ± 0.10% on day 13, indicating almost total tissue repair. This marked difference underscores the superior therapeutic efficacy conferred by the combination of the C-4-N hydrogel and mild photothermal stimulation.

In the diabetes rat model, body weight and blood glucose levels were also concurrently monitored throughout the treatment period (Figure 6D,E). The untreated control group exhibited persistently elevated blood glucose and progressive body weight loss, characteristic features of uncontrolled diabetes in STZ-induced rat models. In contrast, all hydrogel treatment groups showed slower body weight loss and reduced blood glucose. For the C and C-4 groups, the body weight decreased to approximately 204 g, significantly higher than that of the control group of 185.2 ± 3.68 g. Meanwhile, blood glucose decreased to approximately 25 mmol/L, significantly lower than the control group of 33.33 ± 0.12 mmol/L. L-Arg has a clear secretory effect; by stimulating GLP-1, it promotes insulin secretion from pancreatic β-cells and improves glucose tolerance in mice [58]. This effect could be attributed to LA in the hydrogels, which has been shown to enhance endothelial function, insulin sensitivity, and glucose metabolism, thereby aiding in glycemic control and overall diabetic management [59]. Notably, the C-4-N and C-4-N-N groups demonstrated even stronger effects, with slower weight loss and further reduced blood glucose levels. Specifically, these groups maintained body weight at approximately 225 g and achieved blood glucose levels as low as approximately 19 mmol/L. This enhanced efficacy is attributed to the synergistic contribution of CK in the nanoparticles. CK exerts hypoglycemic effects through multiple pathways: stimulating insulin secretion via blockade of ATP-sensitive K^+^ channels, protecting β-cells from apoptosis via SAPK/JNK signaling modulation, enhancing peripheral glucose uptake while suppressing hepatic gluconeogenesis, and reducing inflammation by downregulation of IL-6 and TNF-α and inhibition of NF-κB activation [60]. In summary, the hydrogels exerted systemic glucose-regulatory effects, which significantly contributed to the acceleration of local wound healing.

### 2.7. Histological and Immunofluorescence Analysis

**Figure 7:**
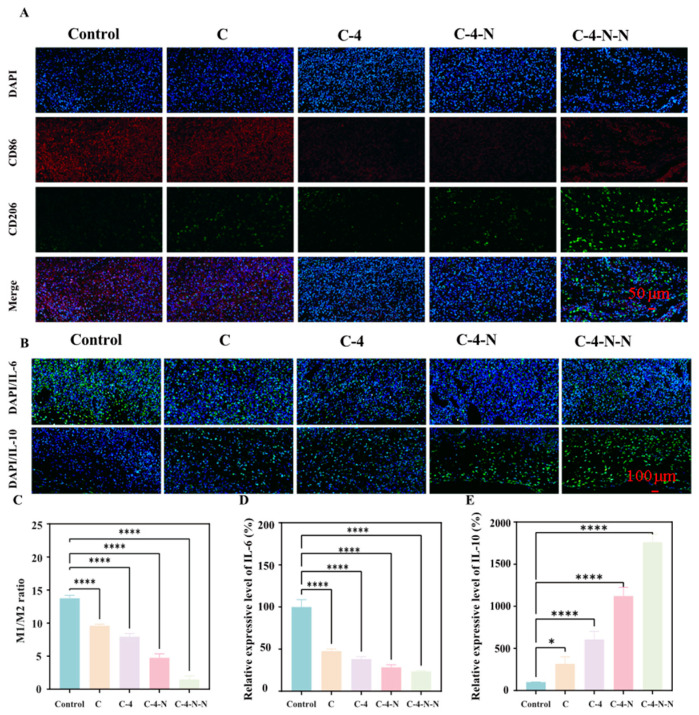
In vivo immunomodulatory effects of the composite hydrogels in diabetic wounds on day 5. (**A**) Immunofluorescence staining of CD86 and CD206. (**B**) Immunofluorescence staining of IL-6 and IL-10. (**C**) Quantitative M1/M2 ratio based on CD86/CD206 fluorescence intensity. Relative expression levels of IL-6 (**D**) and IL-10 (**E**). Sample size *n* = 5. * *p* < 0.05, and **** *p* < 0.0001.

**Figure 8:**
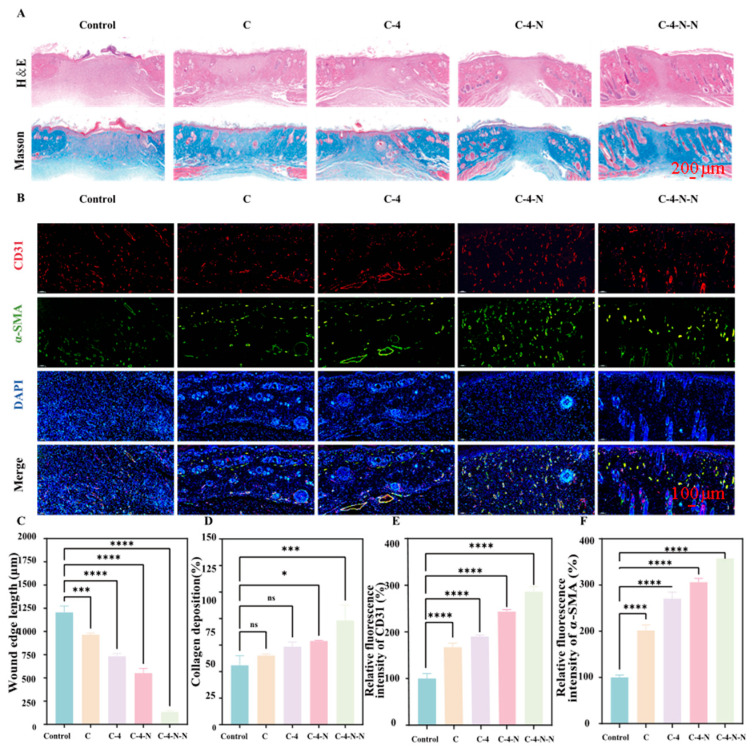
Histological and immunohistochemical analysis of regenerated wound tissues on day 13. (**A**) H&E (**upper**) and Masson (**lower**) staining. (**B**) Immunofluorescence staining of CD31 (green) and α-SMA (red). (**C**) Quantitative wound length (epidermal gap) based on H&E staining. (**D**) Quantitative collagen deposition based on Masson staining. Quantitative fluorescence intensity of CD31 (**E**) and α-SMA (**F**). Sample size *n* = 5. * *p* < 0.05, *** *p* < 0.001, and **** *p* < 0.0001.

To evaluate the in vivo immunomodulatory effects of the composite hydrogels, immunofluorescence assays were performed on skin wound tissue sections collected on day 5 post-wounding. In normal wound healing, this time point corresponds to the peak of the inflammatory phase, however, the inflammatory phase is often prolonged in diabetic conditions, making day 5 an appropriate time point to assess immunomodulatory interventions targeting inflammation resolution. The expression levels of CD86 and CD206 were assessed and shown in Figure 7A,C. Compared with other groups, the C-4-N-N group exhibited significantly reduced CD86 fluorescence intensity and markedly increased CD206 fluorescence intensity. Quantitative analysis revealed that the M1/M2 ratio (13.758 ± 0.35) in the C-4-N-N group decreased by approximately 6-fold compared with the control group (1.48 ± 0.43). These results indicate that the C-4-N-N group effectively promoted the polarization of macrophages toward the anti-inflammatory M2 phenotype, thereby shifting the local immune microenvironment from a pro-inflammatory to a pro-regenerative state. Furthermore, the expressions of key cytokines were evaluated to assess the functional consequences of macrophage polarization. Immunofluorescence staining for the pro-inflammatory cytokine IL-6 and the anti-inflammatory cytokine IL-10 was performed on the same wound tissue sections (Figure 7B,D and Figure 8E). Compared with the control group, all hydrogel-treated groups significantly reduced IL-6 expression and increased IL-10 expression. Notably, the C-4-N-N group exhibited the lowest IL-6 expression levels and the highest IL-10 expression levels among all groups, indicating that the inflammatory response was effectively attenuated while the anti-inflammatory response was enhanced. Collectively, these results demonstrate that the C-4-N-N group possesses potent immunomodulatory capacity in vivo, promoting M1-to-M2 macrophage polarization and rebalancing the pro-inflammatory/anti-inflammatory cytokine network. This immunomodulatory effect is a key contributing factor to the accelerated wound healing observed in diabetic rats.

To evaluate the quality of wound tissue regeneration, histological and immunohistochemical analyses were performed on wound tissue sections harvested on day 13 post-wounding. As shown in Figure 8A, H&E staining revealed distinct differences in wound closure among the experimental groups. The C-4-N-N group exhibited the shortest wound length, indicating almost complete re-epithelialization. In contrast, the control group showed a persistently wide wound gap with limited epithelial migration. Quantitative analysis (Figure 8C) further confirmed these observations, demonstrating the C-4-N-N group exhibited the wound length of 133.33 ± 13.31 μm, which is one-seventh that of the control (1206.00 ± 56.166 μm). These results indicate that the combination of the C-4-N hydrogel with mild photothermal stimulation (the C-4-N-N group) effectively promotes keratinocyte migration and wound closure.

Masson staining was performed to assess collagen deposition and maturation, which are critical indicators of tissue remodeling (Figure 8A, lower). Blue-stained collagen fibers were more abundant and densely packed in the C-4-N-N group compared with the control group, which displayed sparse and disorganized collagen deposition. Quantitative analysis of collagen-positive areas revealed that the C-4-N-N group exhibited the highest collagen deposition of 78.53 ± 11.69%, which was significantly higher than that of the control group (44.78 ± 7.23%) (Figure 8D). These results demonstrate that the C-4-N-N effectively promotes collagen deposition and wound healing.

The formation of mature blood vessels is essential for supplying oxygen and nutrients to regenerating tissues. Immunofluorescence staining for CD31 and α-SMA was performed to evaluate angiogenesis and vascular maturation (Figure 8B). The C-4-N-N group exhibited the highest density of CD31-positive cells, with significantly stronger fluorescence signals and a denser distribution compared with the other groups, indicating robust neovascularization and the formation of a dense microvascular network at the wound site. Furthermore, α-SMA-positive cells were observed surrounding CD31-positive endothelial cells, and this green fluorescence was more pronounced in the C-4-N-N group, with high intensity and uniform distribution, suggesting the formation of mature, functional blood vessels with intact pericyte coverage. Quantitative analysis of fluorescence intensity confirmed that the C-4-N-N group had significantly higher CD31 and α-SMA expression levels compared with all other groups (Figure 8E,F). These results demonstrate that the C-4-N-N hydrogel not only promotes angiogenesis but also facilitates vascular maturation, which is critical for sustained tissue perfusion and long-term wound healing.

## 3. Conclusions

This study developed a mild heat stimulating and micro-environment reprogramming C-4-N hydrogel via a green synthetic strategy for accelerating diabetic wound healing. The hydrogel was constructed by integrating CK/L-Arg/PPA nanoparticles into a dynamic disulfide network of CS-LA and 4-arm-PEG-SH under UV irradiation without toxic initiators, while the PA self-polymerization was spontaneously triggered by the basic condition induced by L-Arg. The C-4-N hydrogel effectively reprogrammed the diabetic wound microenvironment through three synergistic mechanisms, scavenging ROS, promoting M1-to-M2 macrophage polarization, and enhancing angiogenesis. Upon mild photothermal stimulation at 43 °C, the C-4-N-N hydrogel achieved a near-complete healing rate of 99.49 ± 0.10% on day 13 in the diabetic rat model, accompanied by enhanced re-epithelialization, organized collagen deposition, mature blood vessel formation, and systemic glucose-regulatory effects. Collectively, this green-synthesized, mild heat-stimulating hydrogel establishes a synergistic microenvironment reprogramming therapeutic paradigm that integrates antioxidant, immunomodulatory, and angiogenic functions, offering a promising strategy for chronic diabetic wound management.

This study falls within the realm of basic research; no clinical controlled trials have been conducted to date, and a comprehensive validation of the pharmacokinetic evaluation, long-term safety evaluation, and underlying mechanisms of action has not yet been completed. At this stage, the study still has certain limitations and remains a considerable distance from practical clinical translation. Future studies can address the shortcomings of the current research by incorporating commonly used clinical materials as control groups. By integrating the latest relevant research findings from both domestic and international sources to optimize and refine the overall experimental protocol, the validity and practicality of the research findings can be further validated, thereby laying a theoretical foundation for subsequent clinical translation. Furthermore, as technology advances, there is a growing need for integrated smart diagnosis, treatment, and health monitoring [61]. In future research, we can combine the findings of this study with health monitoring technologies to enable real-time monitoring of wound conditions. This will help bridge the gap between innovation in advanced materials and practical applications in the medical field.

## 4. Materials and Methods

### 4.1. Materials

Chitosan (CS, MW ≈ 1650 kDa, DD ≥ 80%) was purchased from Laizhou Haili Biological Product Co., Ltd. (Laizhou, China). Ginsenoside compound K (CK, purity ≥ 96%) was supplied by Xi’an Juzi Biotechnology and Genetic Engineering Co, Ltd. (Xi’an, China). Alpha-lipoic acid (LA, purity = 99%), N-hydroxy succinimide (NHS, 98%),1-(3-dimethylaminopropyl)-3-ethyl carbodiimide hydrochloride (EDC, 98%), L-Arginine(L-Arg, purity = 99.5%), 4-arm-PEG-SH (purity ≥ 97%), protocatechuic aldehyde (PA, purity ≥ 98%), hydrogen peroxide solution (H_2_O_2_, 30 wt% in H_2_O), acetic acid (CH_3_COOH, purity ≥ 99.8%), ethanol (C_2_H_5_OH, purity ≥ 99.8%) were purchased from Shanghai Macklin Biochemical Co., Ltd. (Shanghai, China). 2′,7′-Dichlorofluorescein (DCFH-DA) was purchased from Beijing Solarbio Science & Technology Co, Ltd. (Beijing, China). AO/EB staining Kit was purchased from Beijing Reagan Biotechnology Co., Ltd. (Beijing, China). All the other chemical solvents and reagents were analytical grade and were used as received. CD86 Polyclonal Antibody (bs-1035r), Anti-Mannose Receptor/CD206 Rabbit pAb (GB113497), Anti-CD31 Rabbit pAb (GB11063), Anti-alpha smooth muscle Actin Mouse mAb (GB121364), DAPI (G1012) were purchased from Servicebio (Wuhan, China).

### 4.2. Preparation of CK/L-Arg/PPA Nanoparticles

L-Arg/PPA nanoparticles were synthesized via an oxidative self-polymerization method. Briefly, 100 mg of L-Arg was dissolved in 1 mL of deionized water, followed by the addition of PA (500 mg). After 24 h of reaction, the solution gradually transitioned from light yellow to dark brown, indicating the formation of L-Arg/PPA nanoparticles. The resulting nanoparticles were collected by centrifugation at 12,000 rpm for 15 min and washed three times with deionized water. The purified L-Arg/PPA nanoparticles were collected via freeze-drying. For drug loading, 10 mL of the L-Arg/PPA nanoparticle solution (1 mg/mL) was mixed with 2 mL of CK solution (5 mg/mL). After stirring for 6 h, the mixture was centrifuged at 12,000 rpm for 15 min, and the final CK/L-Arg/PPA nanoparticles was obtained via freeze-drying.

### 4.3. Synthesis of the CS-LA

The synthesis of CS-LA conjugate was carried out according to an established amidation method with slight modifications [62]. Briefly, 0.5 g of CS was dissolved in 100 mL of 1% (*v*/*v*) acetic acid aqueous solution under stirring for 1 h. Then, 0.5 g of LA dissolved in 60 mL of ethanol was added to the CS solution, and the mixture was stirred for another 1 h. Subsequently, NHS (0.8 g) and EDC (0.8 g) were added to the reaction mixture. The amidation reaction was allowed to proceed for 24 h. After the reaction is complete, transfer the mixed solution into a dialysis bag with a molecular weight cut-off (MWCO) of 8000–14,000 Da; use ultrapure water as the dialysate and perform dialysis under continuous magnetic stirring; replace the water every 3 h for the first 12 h, then every 6 h thereafter; the total dialysis time is 6 days; after filtering through a 0.22-micron filter membrane, freeze-dry the solution.

The lyophilized CS-LA was tested using ANALYZER (CHNS) (Elementar Unicube, Hesse, Germany) to determine the content of elements including C, H, N, and S, and the grafting amount of LA were calculated by the following formula [63].

$$\mathrm{GR}=\frac{{7200\times W}_{S}}{{64\times W}_{C}-{96\times W}_{S}}\times 100\%$$

### 4.4. Preparation of the C-4-N Composite Hydrogels

To prepare the composite hydrogel, a mixture containing CS-LA (5% *w*/*v*), 4-arm PEG-SH (5% *w*/*v*), and CK/L-Arg/PPA nanoparticles (0.6% *w*/*v*). The solution was sonicated for 10 min at room temperature to obtain a homogeneous a precursor solution. The resulting precursor solution was exposed to ultraviolet (UV) light (365 nm, 5.8 W/cm^2^) for 10 min to initiate the thiol-disulfide cross-linking reaction. Upon completion of the reaction, a composite hydrogel was formed, which was named as C-4-N. A hydrogel, using the identical protocol but without CK/L-Arg/PPA nanoparticles, was named as C-4. The hydrogel prepared using the same method but containing only CS-LA was designated C.

### 4.5. Characterization

The microstructure of the nanoparticles was characterized by TEM (TEM, Carl Zeiss, Oberkochen, Germany). The chemical structure of the nanoparticles was confirmed by FT-IR (Nicolet Instrument Corporation, Madison, WI, USA) and ^1^H NMR (Bruker Avance NEO 400 MHz, Bremen, Germany). FT-IR spectroscopy and ^1^H NMR spectroscopy were performed on CS-LA to confirm the chemical shifts. The morphology of the composite hydrogel was characterized using a scanning electron microscope (SEM, Carl Zeiss, Oberkochen, Germany).

### 4.6. Rheology Properties

Rheological measurements were performed on a dynamic rotational rheometer (Anton Paar, MCR302, Graz, Austria). The hydrogel precursor solution was taken to the sample stage and exposed to UV light to form a hydrogel. The strain was set to 1% and the frequency was set to 1 Hz, and the time scan test was carried out. After 1 min, the ultraviolet lamp was turned on to irradiate, and the time-modulus curve was obtained. Self-healing test of C-4-N hydrogels was evaluated according to the publication [64]. C-4-N hydrogel samples with diameter of 20 mm and thickness of 0.5 mm were first prepared, and then the value of the critical strain region was recorded by performing the strain amplitude sweep method. Then, the other hydrogel disks were used to evaluate the self-healing performance by carrying out an alternate step strain sweep test at a fixed angular frequency (1 rad/s).

### 4.7. Adhesion Properties of Hydrogels

The prepared hydrogels were applied to the surfaces of various organs and photographed with a digital camera to visually demonstrate their adhesion properties. In addition, adhesion tests were conducted on pig skin to quantitatively evaluate the hydrogels’ adhesive strength. Before the test, porcine skin was pretreated by removing the grease layer and wiping off the excess grease on the external surface. Apply the hydrogel to the cleaned pig skin, covering an area of 1 cm × 1 cm. The adhesion properties of three groups of hydrogels were characterized by a universal testing machine.

### 4.8. Swelling of the Hydrogel

To evaluate structural stability, the hydrogel was immersed in PBS, incubated at 37 °C, and weighed. The water absorption and swelling rate of the hydrogel was determined by measuring the change in weight before and after immersion.

### 4.9. Release of CK/L-Arg/PPA Nanoparticles

Briefly, 0.6 mg/mL of CK/L-Arg/PPA nanoparticles were incorporated into the hydrogel, which was then sealed in a dialysis bag (molecular weight cut-off: 500 Da). The dialysis bags were immersed in centrifuge tubes containing buffers of different pH values (pH 9, 7.4, and 6). To measure drug release, 1 mL of dialysate was collected at specific time intervals and replaced with an equal volume of buffer to maintain a constant volume. The absorbance of the CK/L-Arg/PPA nanoparticles was measured periodically at a wavelength of 280 nm using a UV spectrophotometer.

### 4.10. Antibacterial

To research the antibacterial effect of composite hydrogels, two types of bacteria, *S. aureus* and *E. coli*, were separately cultured with the hydrogels (at a concentration of 0.1 g/mL). The C-4-N-N group was treated with NIR (2.0 W/cm^2^, 15 min) The co-incubated bacterial solution was collected and diluted 10^5^ times; subsequently, 100 μL of the diluted bacterial solution was applied to a new solid medium. Finally, the solid medium was incubated at 37 °C for 24 h, photographed, and the number of colonies was counted. The morphology of the bacteria after treatment was observed via SEM. The bacteria were treated with glutaraldehyde solution (2.5%) overnight and then dehydrated using different concentrations of ethanol solutions (30%, 50%, 70%, 80%, 90%, 100%). Finally, the obtained solution was dispersed in absolute ethanol to prepare samples.

### 4.11. Cytotoxicity Test

L929 cells were inoculated in 96-well plates (5000 cells per well) and cultured for 12 h to complete adherence to the wall. The original medium was aspirated, conditioned medium obtained by coculturing with sterilized hydrogel for 24 h was added, and incubation was continued for 24 and 48 h. At the end of the incubation, cell viability was measured using a Cell Counting Kit-8 (CCK-8, Abbkine Scientific Co., Ltd., Wuhan, China). Cell Live/Dead Fluorescent Staining. L929 cells were inoculated in 24-well plates (50,000 cells per well) and cultured for 12 h to complete adherence to the wall. The original culture medium was replaced with the conditioned medium and continued to incubate for 24 and 48 h. At the end of incubation, the conditioned medium was removed, and 200 μL of AO/EB was added. After 15 min, the cells were washed once with PBS and observed under a fluorescence microscope and photographed.

### 4.12. Cell Migration

HUVECs were cultured in DMEM-F12 (Grand Island Biological Company, Grand Island, NY, USA) medium. HUVECs were infused into a 6-well plate at a density of 5 × 10^4^ cells per well for cell migration assays, and then the cells were incubated for 24 h. Next, a vertical scratch was made in each well using a 200 μL pipette tip, followed by washed with PBS solution to remove unattached cells. The cells were then cultured with hydrogel extracts. After 0, 12, and 24 h, the cells were observed by a fluorescence microscope.

### 4.13. Detection of Angiogenic Capacity

Thaw the matrix glue in advance and pre-cool the 24-well plate, then dispense 200 μL of matrix glue into each well. After allowing the matrix glue to gel in the cell incubator, human umbilical vein endothelial cells (HUVECs) were seeded onto the matrix gel [65]. The control group was treated with complete medium, while the remaining three groups were treated with aqueous gel extract. Cell culture was then performed. After 8 and 12 h, observations and photographs were taken using a light microscope, and ImageJ 1.52a was used for statistical analysis.

### 4.14. Antioxidant Properties

To evaluate the effect of composite hydrogels on cell activity under oxidative stress conditions, H_2_O_2_ was used to establish an oxidative stress model. Intracellular ROS scavenging experiments were performed by coculture. Briefly, RAW 264.7 cells were inoculated in 6-well plates, and 2 mL of medium was added to each well. The 6-well plate was placed in a humidified incubator at 37 °C with 5% CO_2_. After 24 h of culture, the culture medium was removed. DCFH-DA probe was added in the dark, and the cells were incubated in the dark at room temperature for 20 min. All solution was aspirated, and the cells were washed multiple times with PBS. Next, 500 μL of DAPI was added, and the cells were incubated at room temperature for 5–10 min. The solution was aspirated, the cells were washed with PBS, and then observed and photographed under a fluorescence microscope. The group treated with H_2_O_2_ was a positive control, and the group not treated with H_2_O_2_ and hydrogel was a negative control.

### 4.15. Anti-Inflammatory Properties

To assess the differentiation of RAW 264.7 cells under inflammation conditions. RAW 264.7 cells were co-cultured with hydrogel extract (0.1 g/mL) and 100 ng/mL LPS for 24 h. Cells were fixed (4% paraformaldehyde), permeabilized (0.1% Triton X100) and blocked (3% bovine serum albumin (BSA)), then incubated with primary antibodies (CD86 and CD206) and fluorescently labeled secondary antibodies (Alexa Fluor 488 (GB25303) and Cy3 conjugated Goat Anti-Rabbit IgG (GB21303)), and the nucleus was stained with 4′,6-diamidino-2-phenylindole (DAPI (G1012)). CD86 Polyclonal Antibody (bs-1035r), Anti-Mannose Receptor/CD206 Rabbit pAb (GB113497), DAPI (G1012), Alexa Fluor 488 (GB25303) and Cy3 conjugated Goat Anti-Rabbit IgG (GB21303) were purchased from Servicebio (Wuhan, China). Finally, fluorescence microscopy was used to capture immunofluorescence images (Eclipse C1, Nikon, Tokyo, Japan).

### 4.16. In Vivo Photothermal Effects

To investigate the in vivo photothermal effect, the hydrogels applied to the wounded backs of rats. Under near infrared radiation (808 nm, 1.8 W/cm^2^, 10 min, once daily for 0–5 days), the temperature changes were monitored with an infrared thermal imager (ST9550, AMSRT SENSOR, Hong Kong, China) at predetermined time intervals.

### 4.17. Diabetic Skin Wound Healing Assay In Vivo

All animal experiments strictly followed the Animal Management Rules of the Ministry of Health of the People’s Republic of China and approved by the Institute Animal Ethics Committee of Northwest University (Certificate number: NWU-IACUC-20251203R). The animal strain is SD rats, classified as SPF, weighing approximately 200 g, all male, with a total of 50 animals, purchased from Xi’an Jiaotong University (Xi’an, China). To induce a diabetic rat model, the study involved intraperitoneal injection of a 1% (*w*/*w*) streptozotocin (STZ) solution. For the Creation of the Wound Model: Anesthesia was induced by intraperitoneal injection of 2% sodium pentobarbital, and a full thickness incision (Φ = 8 mm) was created on the back of the rats. For the Treatment Process: Dressings were changed on days 0, 1, 3, 5, 7, 9, 11, and 13, and photographs of the wounds and the rats’ body weights were recorded. In addition, previous studies have reported that blood glucose control is critical for the healing of diabetic wounds; therefore, real-time monitoring of blood glucose levels in diabetic wounds is of utmost importance. This study examined blood glucose levels in rats [66]. Skin tissue samples were collected, fixed with 4% paraformaldehyde solution, and sectioned for staining. IL-6 and IL-10 immunofluorescence staining was used to evaluate the inflammation, and CD31 and a-SMA was used to evaluate blood vessels. Immunofluorescence staining for M1 andM2 macrophages with CD86 and CD206 was performed to evaluate the M1/M2 balance of the wound [67]. Additionally, H&E staining was used to evaluate the general structure and morphology of the wound tissues, Masson’s trichrome staining was used to visualize collagen deposition in the wound healing process. C represents the modified chitosan (CS-LA) group; C-4 represents the composite hydrogel formed by CS-LA and 4-arm-PEG-SH; C-4-N represents the composite hydrogel loaded with nanoparticles; C-4-N-N represents the group in which the nanoparticle-loaded composite hydrogel was irradiated with near-infrared light at a power of 1.8 W/cm^2^ for 10 min, as shown in Figure S4.

### 4.18. In Vivo Evaluation of Systemic Toxicity

At the end of the animal experiments, to further evaluate the biosafety of the hydrogel, we performed histological analysis of major organs such as the heart, liver, spleen, lungs, and kidneys of SD rats after 13 days of treatment (Figure S5). H&E staining results indicated that there was no cumulative toxicity in all groups [68].

### 4.19. Statistical Analysis

The results of statistical analysis were expressed as the mean ± standard deviation. The experimental results were analyzed and processed using ImageJ 1.52a software. All data are expressed as the mean ± standard deviation (SD). Differences between groups were assessed using GraphPad Prism 10.1.2 software, with statistical significance indicated by * *p* < 0.05, ** *p* < 0.01, *** *p* < 0.001, and **** *p* < 0.0001 [69].

## Data Availability

The original contributions presented in this study are included in the article/Supplementary Material. Further inquiries can be directed to the corresponding authors.

## Supplementary Materials

The following supporting information can be downloaded at: https://www.mdpi.com/article/10.3390/gels12060542/s1. Table S1—Grafting efficiency of CS-LA. Table S2—Grafting efficiency Nanoparticle Stability. Figure S1—Macroscopic self-healing of hydrogels. Figure S2—The hydrogel was adhered to various tissues. Figure S3—Detection of radical scavenging capacity. Figure S4—A Thermal imaging photograph of a rat wound. B. Photothermal cycle curves at the wound site. C. Photothermal curves on days 0 and 1 after dressing application. Figure S5—Effects of hydrogels on histology of major organs in rats.

## Abbreviations

The following abbreviations are used in this manuscript:

CS	Chitosan
LA	Alpha-lipoic acid
EDC	1-Ethyl-3-(3-dimethylaminopropyl) carbodiimide hydrochloride
NHS	N-hydroxy succinimide
FT-IR	Fourier transform infrared
^1^H NMR	^1^H Nuclear magnetic resonance
G′	storage modulus
G″	loss modulus
*S. aureus*	Staphylococcus aureus
*E. coli*	Escherichia coli
H&E	Hematoxylin and Eosin
Masson	Masson’s Trichrome Staining
