# Supplementary material for: Mild Heat Stimulating and Microenvironment Reprogramming Hydrogel for Accelerating Diabetic Wound Healing
Source: Gels. 2026 Jun 17;12(6):542. doi: 10.3390/gels12060542 (PMC13298256; doi:10.3390/gels12060542)
Supplement: Supplementary file 1 [file gels-12-00542-s001.zip › gels-4370116-supplementary.pdf]

Supporting information

Mild heat stimulating and microenvironment reprogramming hydrogel for accelerating diabetic wound healing

Xueting Xiao[1, 2], Yannan Liu[1, 2], Dan Li[1, 2], Lebin Wang[1, 2], Zirui Hu[1, 2], Xinliang Xing[1, 2], Yali Ding[1, 2], Xurun Wang[1, 2], Ruifan Zhang[1, 2], Jing Yang[1, 2, *], Xiaoxuan Ma[1, 2, *]

1 Engineering Research Center of Western Resource Innovation Medicine Green Manufacturing, Ministry of Education, School of Chemical Engineering, Northwest University, Xi'an 710127, China.

2 Shaanxi Key Laboratory of Biomaterials and Synthetic Biology, Shaanxi R&D Center of Biomaterials and Fermentation Engineering, School of Chemical Engineering, Northwest University, Xi'an 710127, China.

[*]Correspondence: yangjing2018@nwu.edu.cn (J.Y.); xiaoxuanma@nwu.edu.cn(X.M.)

Table S 1 Grafting efficiency of CS-LA

	C（%）	H（%）	N（%）	S（%）	Grafting rate（%）
CS	41.24	6.68	7.47	0	0
CS-LA	39.49	8.26	5.41	9.68	43.61

Table S2 Grafting efficiency Nanoparticle Stability

Time（h）	Size（nm）	Zeta potential（mV）
0	200.64 ±12.86	-32.5 ± 0.77
24	218.10±1.38	-22.9 ± 0.32
48	238.30±25.22	-13.36 ± 0.04

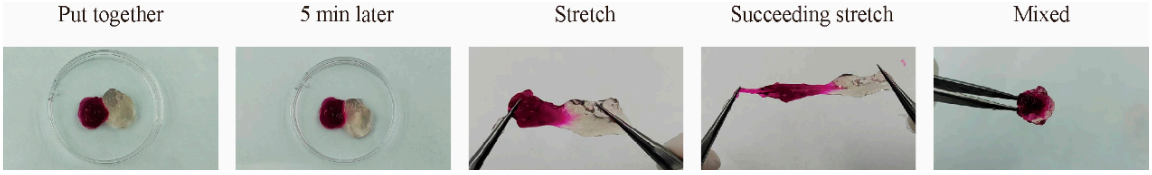

Figure S1 Macroscopic self-healing of hydrogels.

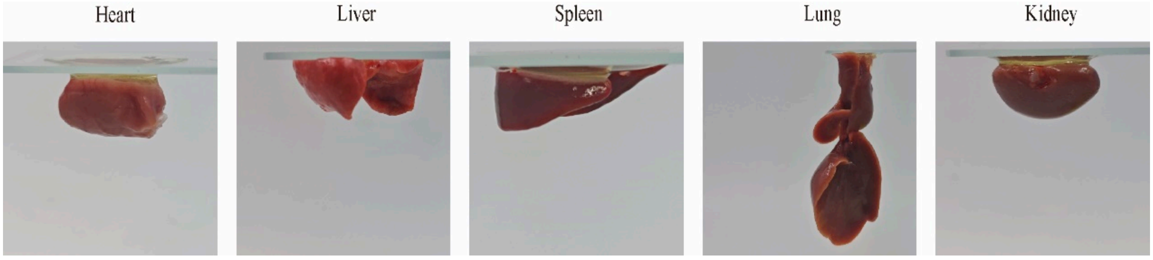

Figure S2 The hydrogel was adhered to various tissues.

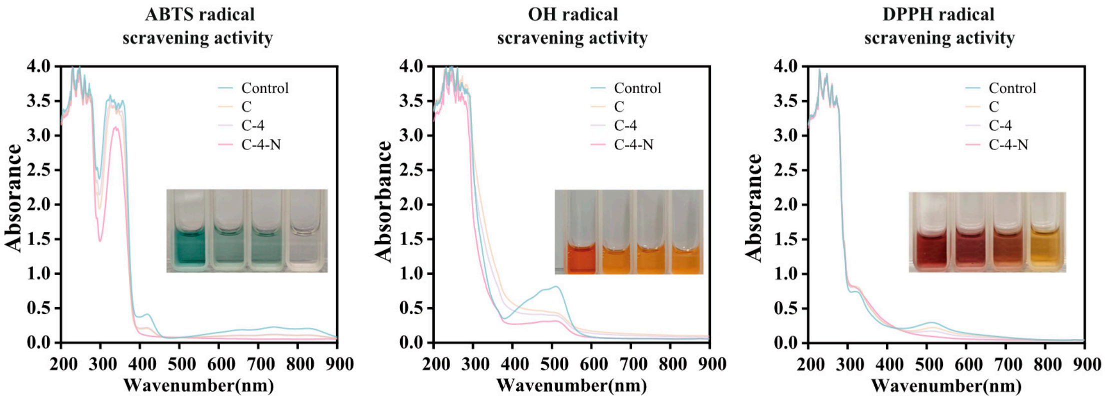

Figure S3 Detection of radical scavenging capacity

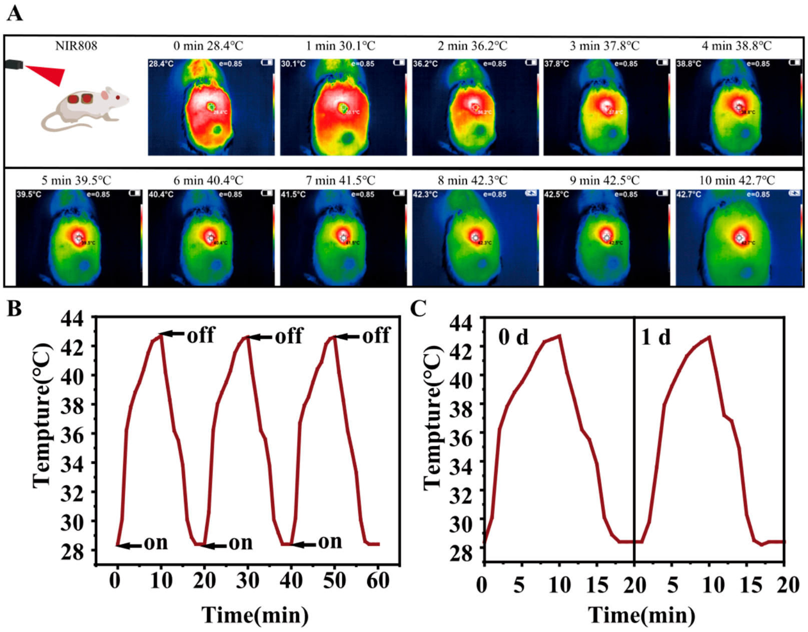

Figure S4 A Thermal imaging photograph of a rat wound. B. Photothermal cycle curves at the wound site. C. Photothermal curves on days 0 and 1 after dressing application.

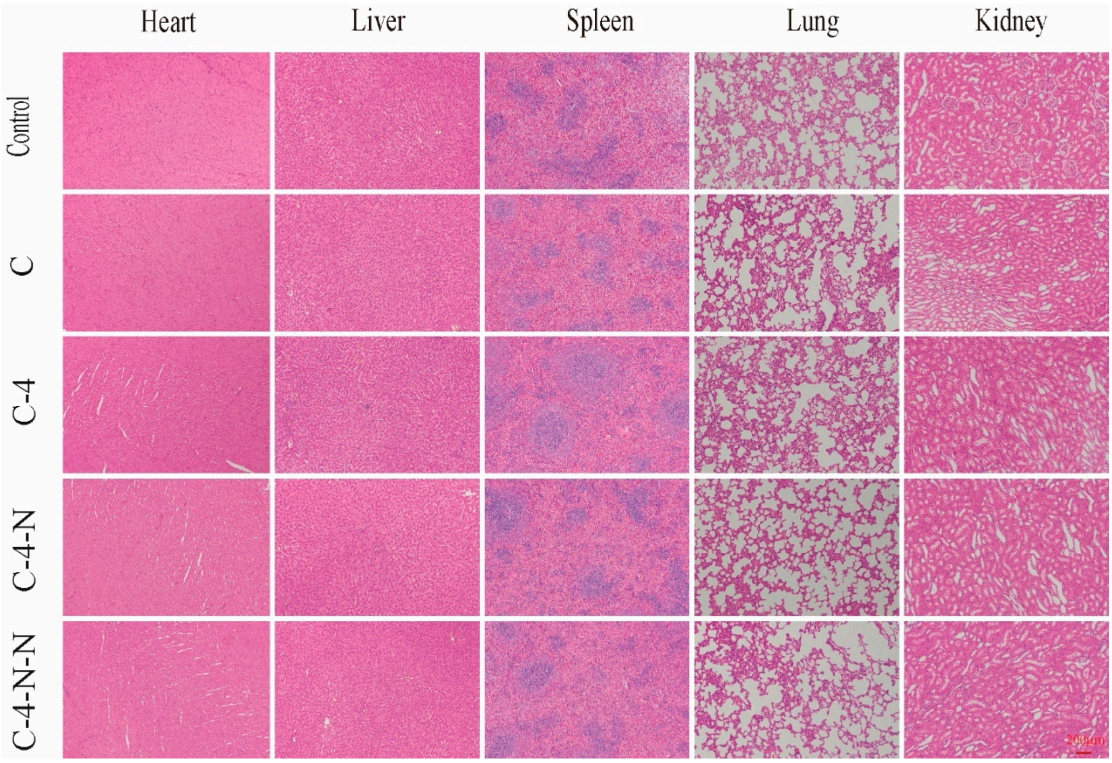

Figure S5 Effects of hydrogels on histology of major organs in rats
